# Book Notices

**Published:** 1896-09

**Authors:** 


### Book Notices.

SPECTACLES AND EYEGLASSES: THEIR FORMS, MOUNTINGS AND PROPER ADJUSTMENT. By R. J. Phillips, M. D., Ophthalmic Surgeon to the Presbyterian Hospital in Philadelphia, etc. Second edition revised, with forty-nine illustrations. Price, $1. P. Blackinston, Son & Co. Publishers, Philadelphia.

This excellent book has now reached its second edition, and is no doubt the best book on the subject of the proper selection and fitting of eyeglasses. It should be in the hands of every student of ophthalmology and every ophthalmic surgeon.

---

DIET FOR THE SICK. Contributed by Miss E. Hibbard, Principal of Nurses' Training School, Grace Hospital, Detroit, and Mrs. Emma Drant, Matron of Michigan College of Medicine Hospital, Detroit. Second Edition, Enlarged. Limp

Cloth, 16mo., 100 pages. Price 25 cents, postpaid. Detroit, Mich.: The Illustrated Medical Journal Co., 1896.

In this little book there is, besides the useful formulæ for "Sick Dishes," foods and cooling drinks for convalescents, quite complete Diet Tables for use in Anæmia, Bright's Disease, Calculus, Cancer, Chlorosis, Cholera Infantum, Constipation, Consumption, Diabetes, Diarrhœa, Dyspepsia, Fevers, Gout, Nervous Affections, Obesity, Phthisis, Rheumatism, Uterine Fibroids. It also gives various nutritive enemas. The physician can use it to advantage in explaining his orders for suitable dishes for his patient, leaving the book with the nurse.

---

THE MULTUM IN PARVO REFERENCE AND DOSE BOOK. By C. Henri Leonard, M. A., M. D., Professor of the Medical and Surgical Diseases of Women, Detroit College of Medicine. Flexible leather, 143 pages, price 75 cents. Detroit, 1896: The Illustrated Medical Journal Co., Publishers.

This is a recent edition of the Dose Book, of which the title page informs us some forty thousand copies have been issued. The present edition is printed on very thin paper, and is bound in red leather, round corners, so as to make it specially light and handy for the pocket; the weight is not two and a half ounces. Besides the doses of some 3,500 preparations being given, it has numerous tables, such as the solubility of chemicals, pronunciation of medical proper names, poisons and their antidotes, incompatibles, tests for urinary deposits, abbreviations, table of fees, etc. It will be found a handy pocket companion.

---

TWENTIETH CENTURY PRACTICE. AN INTERNATIONAL ENCYCLOPEDIA OF MODERN MEDICAL SCIENCE. By leading authorities of Europe and America. Edited by Thomas L. Stedman, M. D., New York City. In twenty volumes. Volume VIII. "Diseases of the Digestive Organs." New York: William Wood and Company. 1896.

The publishers have found it necessary to issue Volume VIII out of its regular order, as was the case with Volume VI. Volume VII will be the next volume to appear, upon publication of which the series will be completed as far as Volume VIII.

The present volume treats entirely of diseases of the digestive organs, and has been prepared by eight different authors, four of whom are Americans, and four Germans. The opening chapter, by Drs. Johann Mikulicz and Werner Kummel, of Breslau, deals with the various diseases of the mouth. Dr.

Reginald H. Fitz, of Boston, contributes a chapter of twenty-eight pages on diseases of the œsophagus. The most valuable part of the book is the 249 pages given to diseases of the stomach. This chapter is by Dr. Max Einhorn, of New York, and for careful and thorough investigation, especially of the practical points in the diagnosis and treatment of the affections of the stomach, it deserves especial mention. Dr. H. Leo, of Bonn, contributes a very interesting chapter on the diseases of the pancreas. Another very important chapter is that on diseases of the peritoneum, by Dr. B. Farquhar Curtis, of New York.

The subject of animal parasites and the diseases caused by them, has received due attention at the hands of Dr. J. Ch. Huber, of Memmingen, Bavaria.

Dr. James M. French, of Cincinnati, contributes the last chapter in this volume, on the subject of treatment of the diseases caused by animal parasites.

Taking this volume as a whole, it possesses unusual merit. The subjects are all practical ones, and the contributors have given to these subjects the careful attention their importance demands. The volume is illustrated by over one hundred original engravings. S. E. H.

---

MEDICAL GYNECOLOGY. A Treatise on the Diseases of Women, from the Standpoint of a Physician, by Alexander J. C. Skene, M. D., Professor of Gynecology in the Long Island College Hospital, Brooklyn, New York; formerly Professor of Gynecology in the New York Post-Graduate Medical School; Gynecologist to the Long Island Hospital; President of the American Gynecological Society in 1887, etc., etc., etc. In one large octavo volume of 530 pages, with illustrations. Price, cloth, $5. D. Appleton & Co., publishers, 76 5th Ave., New York City. 1895.

Dr. Skene's book on the diseases of women, which has now been before the profession for some years, is a very popular one and has fully established his reputation as a medical writer and teacher. The present work is one quite out of the ordinary as it deals only with the medical aspect of gynecological practice, leaving out entirely the surgical. Whether this plan is any advantage even to the general practitioner, we are not prepared to say. The subjects discussed are all important ones to the physician, and no one can read the book and not be greatly benefited thereby.

The arrangement is in three parts. Part I. deals with the primary differentiation of sex, development and growth during early life, and the conditions favorable to the evolution of normal organization and the attainment of a healthful puberty. This covers a very important period in life, involving, as it does, the period of growth and development; the educational, mental and physical, period; and the time when the transition is made from girlhood to womanhood. The various influences that may prevent a perfect development of the body and mind and interfere with the establishment of a healthful puberty, are carefully discussed and proper rules are laid down for the correction or prevention of these unfavorable influences. Part II. treats of the characteristics of sex, the predisposition to particular diseases, and the causes of certain affections peculiar to women. In this part of the book is considered the functional and organic diseases common to the period of functional activity, the diseases incident to the child-bearing period. The inflammatory affections of the pelvic organs, diseases of the external genital organs, of the urinary organs, mammary glands, uterine fibromata, uterine displacements, the various nervous affections dependent upon or associated with disease of the pelvic organs, etc., are considered in this part of the book.

Part III. discusses the menopause and the diseases most likely to occur after the period of functional activity has passed. The author's chapter on the menopause is one of the best in the English language. It is short, covering only twelve pages, but is thoroughly practical, and will be found eminently satisfactory to the practicing physician. H.

